# Backstroke to Breaststroke Turning Performance in Age-Group Swimmers: Hydrodynamic Characteristics and Pull-Out Strategy

**DOI:** 10.3390/ijerph18041858

**Published:** 2021-02-14

**Authors:** Phornpot Chainok, Leandro Machado, Karla de Jesus, J. Arturo Abraldes, Márcio Borgonovo-Santos, Ricardo J. Fernandes, João Paulo Vilas-Boas

**Affiliations:** 1 Faculty of Sport Science, Burapha University, Chonburi 20131, Thailand; 2 Centre of Research, Education, Innovation and Intervention in Sport (CIFI2D) and Porto Biomechanics Laboratory (LABIOMEP-UP), Faculty of Sport, University of Porto, 4099-002 Porto, Portugal; lmachado@fade.up.pt (L.M.); karla_de_jesus@yahoo.com.br (K.d.J.); marcio.labiomep@fade.up.pt (M.B.-S.); ricfer@fade.up.pt (R.J.F.); 3 Faculty of Physical Education and Physiotherapy, Federal University of Amazonas, Amazonas 69067-005, Brazil; 4 Faculty of Sport Science, University of Murcia, 30720 San Javier, Spain; abraldes@um.es

**Keywords:** swimming, hydrodynamics, drag, strategy, age group

## Abstract

We compared the hydrodynamic characteristics and pull-out strategies of four backstroke-to-breaststroke turning techniques in young swimmers. Eighteen 11 and 12-year-old swimmers participated in a 4 week intervention program including 16 contextual interference sessions. The hydrodynamic variables were assessed through inverse dynamics, and the pull-out strategy kinematics were assessed with tracking markers followed by 12 land cameras and 11 underwater cameras. Swimmers randomly completed sixteen 30 m maximal backstroke-to breaststroke-open, somersault, bucket and crossover turns (four in each technique) with a 3 min rest. The data showed higher drag force, cross-sectional area and drag coefficient values for the first (compared with the second) gliding position. The crossover turn revealed the highest push-off velocity (2.17 ± 0.05 m·s^−1^), and the somersault turn demonstrated the lowest foot plant index (0.68 ± 0.03; 68%), which could have affected the first gliding, transition and second gliding depths (0.73 ± 0.13, 0.86 ± 0.17 and 0.76 ± 0.17 m). The data revealed the consistency of the time spent (4.86 ± 0.98 s) and breakout distance (6.04 ± 0.94 m) among the four turning techniques, and no differences were observed between them regarding time and average velocity up to 7.5 m. The hydrodynamic characteristics and pull-out strategy of the backstroke-to-breaststroke turns performed by the age group swimmers were independent of the selected technique.

## 1. Introduction

Turning performance is determined by the efficiency of changing direction while swimming between the turn-in and turn-out phases. Consequently, swimmers should approach the wall by maintaining speed without compromising the ability to turn and push off the wall as powerfully as possible (allowing the highest wall-out velocity with the least possible drag) [1,2,3]. The swimming-related literature emphasizes that the total turning performance results from increased turn-out efficacy throughout the push-off, glide and swimming resumption phases [4,5,6]. In fact, the optimized performance should derive from a balance between promising hydrodynamic propulsion and minimizing hydrodynamic drag [4,7].

Theoretically, swimmers can improve their turn-out performance by improving their underwater gliding efficiency, both minimizing drag and optimizing underwater timing and distance [6]. When gliding, passive drag (Dp) is mainly determined by the swimmer’s body shape, velocity and depth [8,9,10]. The difference in the drag coefficient (C_D_) can be attributed to varying body dimensions or to changes in body position [11,12]. Dp can be experimentally assessed by towing a swimmer in a fixed position [4,13] or through inverse dynamics [14] and numerically using computer fluid analysis (CFD) [10,15,16]. The Dp during the breaststroke underwater action was investigated using inverse dynamics, and it was observed that the drag force (D), drag coefficient (C_D_) and cross-sectional area (S) of the first gliding position were lower than those of the second gliding path [14,16]. However, none of these questions have been addressed in the backstroke-to-breaststroke turning action out of the wall. In this specific turn, swimmers adopt a pull-out strategy that is divided into four phases: first gliding, a transition phase (where an underwater upper and lower limb action takes place), second gliding and transition to the surface [14,15,17].

Gliding performance depends on the initial velocity, as well as the deceleration magnitude and its duration [6]. Furthermore, optimizing gliding distance and time by maximizing the velocity has also been suggested [18]. For instance, if the underwater lower limb action is initiated too early, resistance increases, slowing the swimmer prematurely. Conversely, by gliding for too long before the underwater lower limb action, a swimmer will decelerate to less than the race pace, wasting energy returning to free swimming speed [4,16]. However, optimal timing of the pull-out strategy depends on each individual, since it is related to the time taken to reach the aimed competitive swimming velocity. Since no studies are available that focus on analysing backstroke-to-breaststroke turning performance in young swimmers, considering an integrated multifactorial approach, we aimed to compare the hydrodynamic characteristics and pull-out strategies regarding the turn-out performance of four backstroke-to-breaststroke turn techniques in certain age groups of swimmers.

## 2. Materials and Methods

### 2.1. Experimental Approach

**Figure 1:**
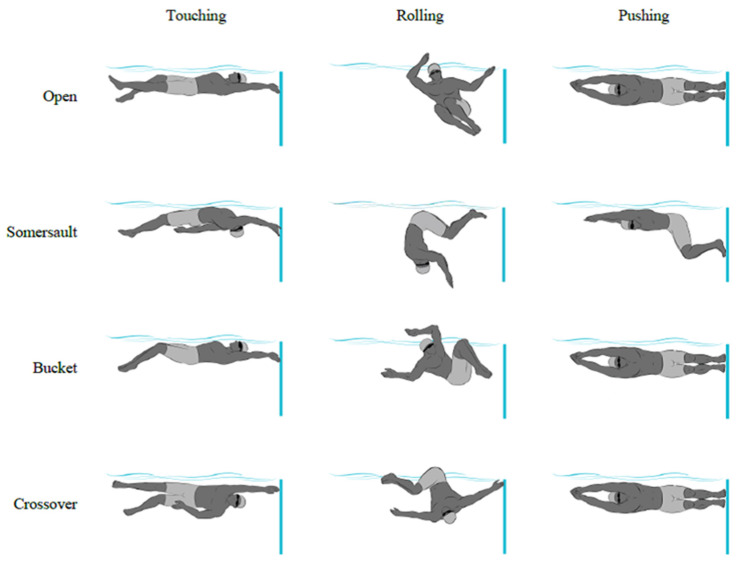
Representation of a swimmer’s body orientations during the rotation and push-off phases for the four studied backstroke-to-breaststroke turning techniques.

The key mechanical features of the four studied backstroke-to-breaststroke turn techniques, considering the hydrodynamics and pull-out strategy, were obtained after intervention training sessions. Those key mechanical features were described and compared using selected kinematic and hydrodynamic factors. Four backstroke-to-breaststroke turns were identified, based on FINA rules SW 6.5 and 9.4 and the complex movement variations that specify body configuration and orientation in the rotation and push-off phases (Figure 1). A 4 week intervention program of 16 systematically increasing contextual interference sessions (40 min each) was conducted in a 25 m (1.90 m deep) indoor pool. Subjects were assigned to train under this program and the practice difficulty progressively increased, with appropriate challenges beyond their skill levels [19], to facilitate learning and improve performance [20]. Coaches were asked to allow swimmers to avoid intense efforts and substantial changes in training, dietary and sleep regimes during the experience period, particularly in the 48 h before each instance of data collection.

Swimmers followed a block-type schedule plan from the first to the fourth practice sessions (each focusing on a different turning technique), and a serial schedule was followed from the fifth to the eighth session (the A, B, C and D series were repeated for 10 min). A serial schedule was also followed from the ninth to the twelfth session (the 5 min series of A, B, C and D were repeated twice). Then, a random schedule was followed from the thirteenth to the sixteenth sessions, with an equal number of trials for each technique. Following the intervention period, swimmers randomly performed twelve repetitions, those being three repetitions of each turning technique (with 3 min of rest between trials). The trials started and finished from the middle of the pool, with swimmers performing turns in and out of the wall at maximum speed until the 15 m reference. An experienced researcher observed every turn and asked swimmers to repeat those not properly performed. The average values (from the three trials) obtained for each selected variable per turning technique were taken for posterior analysis.

### 2.2. Participants

Eighteen age group swimmers (10 males and 8 females) participated in the study. Their main anthropometric, performance and training background characteristic means and standard deviations values were (for males and females, respectively) as follows: 12.45 ± 0.16 and 11.71 ± 0.18 years of age, 48.81 ± 3.57 and 43.47 ± 3.40 kg of body mass, 19.65 ± 0.77 and 18.97 ± 0.90 kg/m^−2^ body mass index, 15.93 ± 1.81 and 18.06 ± 1.41% of fat mass, 158.50 ± 3.89 and 149.28 ± 2.71 cm in height, 157.82 ± 4.06 and 151.00 ± 2.67 cm arm span, 183.73 ± 5.60 and 192.38 ± 5.45 s 200 m individual medley short course best performance (representing 61.89 ± 8.69% and 67.56 ± 6.19% of the world junior record) and 3.56 ± 1.43 and 3.12 ± 1.13 years of competitive experience, and both were in Tanner stages 2–4. Swimmers, coaches and parents were informed of the investigation benefits and risks before signing an informed consent form to participate. The study was approved by the ethics board of the institution (code n° CEFADE 08.2014), and all procedures were in accordance with the Declaration of Helsinki.

### 2.3. Measurements

**Figure 2:**
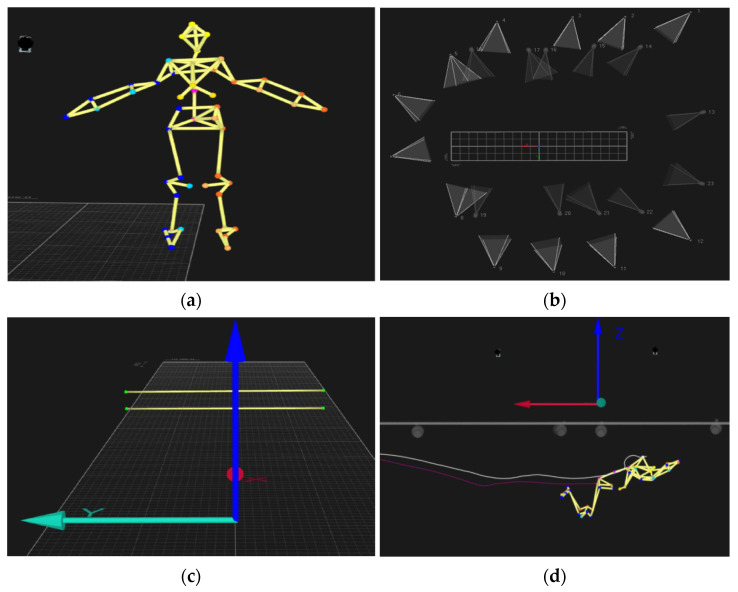
Kinematic data collection and data processing for (**a**) the full-body marker setup, (**b**) motion capture camera positioning, (**c**) referential system origin using four points of reflective markers and (**d**) sacrum and head kinematic traces during the pull-out phase.

Subjects were photographed using scaled photographs [11,14] at a height of 3 m (measured from the ground reference plane) in the first and second gliding positions for S assessment using planimetry [14]. Kinematic data were recorded by automatically tracking 51 spherical retroreflective markers (see Figure 2a panel) with a motion capture set-up that included 12 stationary overwater cameras and 11 underwater cameras (Oqus 3 and 4 series, Qualisys, Gothenburg, Sweden; see the panel in Figure 2b) sampling at 100 Hz. Ten land-based system cameras were mounted along two opposite swimming pool lateral sides (covering the 15 m mark from the wall), and two others were positioned perpendicularly. Nine underwater cameras were also placed along the two opposite lateral sides of the pool just below the water surface (with the respective lenses focusing on the swimmer’s trajectory), and the remaining two cameras were sitting at the bottom of the pool facing upward [21]. No camera was coplanar, and the wand calibration followed the three consecutive steps employed in processing and acquisition: underwater, above water and combined (merging the underwater and above-water views).

Calibration was first performed with a static L-frame (positioned 7.5 m from the wall). Then, wand dynamic calibration was conducted using an L-shaped reference structure and moving a wand with two markers of 0.7495 m of interpoint distance (according to the manufacturer recommendations). Wand dynamic calibrations were performed separately underwater and overwater, and they were combined by merging a land-based and underwater system using dual media wand movements [21]. A calibrated volume of ~30 m^3^ and locations of 10 m (X, pointing horizontally and in the forward motion direction), 2.0 m (Y, horizontally and laterally toward the right of the swimmer) and 1.5 m (Z, vertically) were obtained. The origin of the referential system was set by using four points of reflective markers at the wall (corresponding to a position 7.5 m away from the wall), and the calibration mean precision value obtained was ~0.79 mm. Marker reconstruction accuracy reached 93.2%.

### 2.4. Data Processing

The hydrodynamic variables were assessed through inverse dynamics, considering D, C_D_ and S as previously proposed [14,16]. D was extracted from the relationship between the swimmer’s body mass (m, with the added mass effect not being considered) and acceleration (a) for the first and second glides:

(1) D=m a

**Figure 3:**
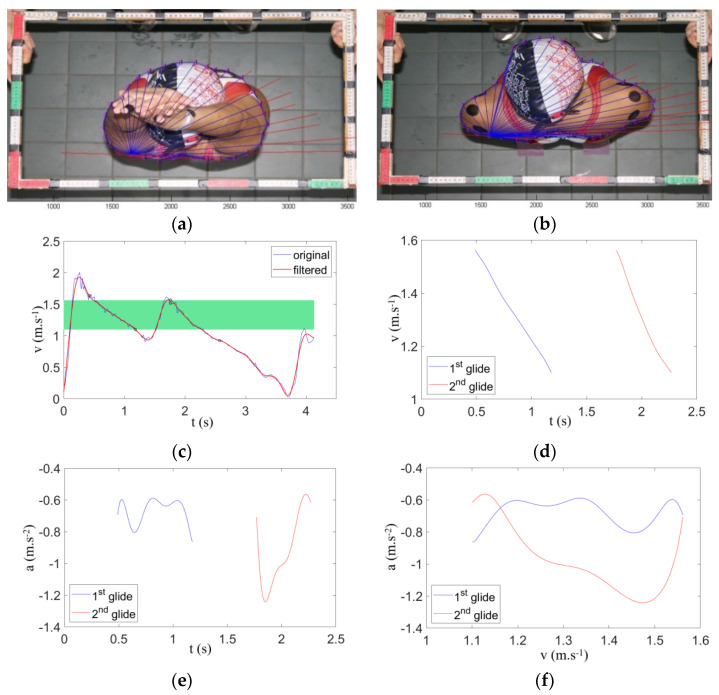
Hydrodynamic data collecting and processing. Body surface area was determined using the first (**a**) and second (**b**) gliding positions, and the v(t) data (**c**) was filtered with a 3 Hz cut-off low-pass fourth order Butterworth filter. The v(t) curve range of two successive gliding phases (**d**), the acceleration-to-time curve (a(t)) (**e**) and the acceleration-to-velocity curves ((a(v)) (**f**) are also displayed.

The acceleration-to-time curve (a(t)) of the sacrum reflective marker was assessed during the first and second glides of the pull-out phase through numerical differentiation of the v(t) curve (filtered with a fourth-order Butterworth filter). The C_D_ for each gliding was calculated using the following transformation:

(2) $$C_{D}=2\;D/\rho \;S\;v^{2}$$

where D is the measured drag, ρ represents the water density (1000 kg·m^−3^), S is the typical cross-section frontal area surface of the first and second gliding positions and v is the swimmer velocity relative to the flow [14,16]. S was directly measured through planimetry using the scaled photograph technique [11,14,16] and was computed in MATLAB^®^ 2014a (The Mathworks, Inc., Natick, MA, USA) as the summation of the triangles’ areas [14]. The S digitizing reliability was evaluated from one randomly selected photograph of a swimmer digitized 10 times in each gliding position. The intraclass correlation coefficient (ICC) was 0.99. The mean values of the three independent digitizing trials were selected (panels in Figure 3a–f).

To determine the turn-out performance, it was necessary to examine the variation and relationships of the featured variables with factors possibly affecting the response to pull-out performance. Qualisys Track Manager (Qualisys, Gothenburg, Sweden) software was used to acquire the 3D kinematic data (see Figure 2d), which was then imported into the signal processing software (Acqknowledge v.3.9.0, BIOPAC Systems Inc., Santa Barbara, CA, USA). Each individual variable was digitally filtered with a 6 Hz cut-off digital filter (FIR, Window Blackman, 61 dB) to minimize artifact noise [22]. The kinematic variables selected to characterize the pull-out strategy are described in Table 1.

### 2.5. Statistical Analysis

After applying a Shapiro–Wilk normality test, mean and standard deviation computations for descriptive analysis were obtained for all variables. Sphericity was verified using the Bartlett test before using repeated ANOVA measures to detect any main effect of the hydrodynamics characteristics, pull-out strategy or the four backstroke and breaststroke turning techniques. Provided that a significant effect was found, Bonferroni post hoc analysis was conducted for each pairwise comparison. To provide an unbiased estimate of the population effect size, a partial omega squared (ω^2^) measurement was adopted [23] and classified as small (<0.06), moderate (0.07–0.14) or large (>0.14) [24].

## 3. Results

The S of the second gliding was higher than the first gliding (584.67 ± 1.14 vs. 632.18 ± 0.08 cm^2^, *p* = 0.028). The other hydrodynamic characteristics of the first and second gliding positions at the open, somersault, bucket and crossover backstroke-to-breaststroke turning techniques are displayed in Table 2. Even if the gliding velocities were similar in all turns, the second gliding displayed higher D and C_D_ values compared with the first one. The results demonstrate that there were no main effects of the turns on S, D and C_D_ among the four turning techniques.

The kinematics and pull-out variables among the four backstroke-to-breaststroke turning techniques are displayed in Table 3. Regarding the three examined components of the push-off phase, statistical analysis highlighted the main effect of the turns on the push-off velocity, with the highest value occurring in the crossover turn. There were no differences for the tuck index among the four turning techniques. The foot plant index decreased from the somersault turn to the open, bucket and crossover turns. For the first gliding phase, it was also observed that the depth was higher in the somersault turn than in the bucket, crossover and open turns. An effect of the turns on the transition phase between the two gliding phases (the underwater upper and lower limb actions) was observed only for the gliding depth. Post hoc analyses demonstrated that the transition depth was higher in the somersault turn than in the bucket, crossover and open turns.

In the second gliding phase, the main effect of the turning techniques was only observed with the depth. Post hoc analyses demonstrated that the value for the second gliding depth was highest in the somersault turn. Values for the second gliding depth were highest for the somersault turn, followed by the bucket, crossover and open turns. The turning techniques did not elicit changes in the breakout distance, breakout time, velocity at breakout or average pull-out velocity. Notably, the time to 7.5 m was not influenced by different backstroke-to-breaststroke turning techniques and pull-out strategies.

## 4. Discussion

The purpose of this study was to compare the hydrodynamic characteristics and the pull-out strategies during turn-out performance of certain age group swimmers when performing four backstroke-to-breaststroke turning techniques. Any differences in time and average velocity to 7.5 m, as well as in the pull-out strategy, after a 4 week intervention program among the open, somersault, bucket and crossover turns were not observed. Contrary to our expectations, the data did not allow for classifying any of the turning techniques as the most effective in terms of turn-out performance (considering hydrodynamic characteristics and pull-out strategy variables). However, there are possible explanations for the obtained results, allowing one to better understand the hydrodynamic characteristics and pull-out strategies used by young swimmers.

Our swimmers’ S values in both the first and second gliding positions were relatively low when compared with the data from national level swimmers (740.42 ± 101.89 and 784.25 ± 99.62 cm^2^) [14]. Regarding the hydrodynamic characteristics, (i.e., D, C_D_ and v), no differences were found among the four studied turns in the two gliding positions. Our findings confirmed the previous results, which suggested that D was associated with anthropometric differences with respect to age and body alignments [14,25]. In the literature, push-off propulsion optimization and pull-out strategy have clearly demonstrated their influence for improving turn performance, since kinematic factors (like tuck index, foot plant position and push-off velocity) play critical roles and directly affect pull-out performance [5]. Theoretically, there are two determining factors that directly affect glide performance: the initial push-off velocity and hydrodynamic drag (which acts to decelerate the swimmer) [9]. We observed higher values for the average push-off velocity when swimmers performed the crossover turn, with data presenting evident similarities with previous studies in age group swimmers that performed butterfly and breaststroke open turns and front crawl tumble turns (2.00 ± 0.20, 2.01 ± 0.21 and 2.01 m·s^−1^, respectively) [26,27,28], but the average push-off velocity was higher when compared with the backstroke turn (1.70 ± 0.30 m·s^−1^) [1].

The higher push-off velocity values observed in the crossover turn could be due to the rotational skills of [3] and lateral body positioning during push-off by the swimmer (considered as more hydrodynamic than the prone position) [29]. These findings should be related to the kinematic factors of the foot plant position that differed among the four turning techniques; the somersault turn tended to display a slightly higher foot plant index than any other turns and also compared with the data reported before (0.40 m and 0.45 m) [5,30]. However, there was no difference in the tuck index among the four studied turning techniques. In the current study, the mean distance of the hip from the wall was ~74% (71–76%) of the length of the swimmer’s lower limbs, which was slightly higher than the values reported before in the breaststroke and backstroke turns (58% and 60%), also for the age group swimmers [1,27].

As was already shown, the pull-out strategy optimization can substantially impact swimming turn performance, with a properly executed streamlined posture, gliding depth and optimal underwater lower and upper limb action timing and distance being key factors for turn-out performance [6,12]. The current study provides additional evidence for the glide depth during pull-out importance. The first gliding, transition and second gliding depth values were slightly higher in the somersault than in any other backstroke-to-breaststroke turn, making it possible to assume that the foot plant position during push-off could be responsible for the glide depth path. Moreover, it was suggested before that swimmers should achieve a ~0.40–0.60 m glide depth to obtain maximum drag reduction benefits at fast exertions, suggesting that the values observed for the somersault turn might not be advantageous. Indeed, no differences in final turning performance were observed.

Choosing the correct gliding duration and distance, as well as a proper lower limb action timing, for maximizing velocity should be an individual strategy. The current data showed that for all the studied turning techniques, swimmers spent ~1.21–1.34 s covering the 2.41–2.60 m first gliding distance before initiating the transition, corroborating the values proposed by Lyttle et al. [4] (which also did not find any differences between lateral and ventral gliding positioning). An important piece of feedback is that swimmers should use an approximately ~0.4–1.0 m glide depth and wait ~1 s before initiating underwater lower limb action [4,31]. In fact, if it is initiated too early, the resistance will increase, slowing down the swimmer prematurely [4]. Moreover, concurrent with a higher S, D and C_D_ in the second gliding (than in the first position), there was a tendency for the swimmer’s average velocity to decrease, in line with the findings of Termin and Pendergast [32] that the average velocity did not increase due to the upper and lower limb actions during the transition phase.

Nonetheless, in spite of the current study’s interesting findings, there are some limitations that should be considered and addressed in future research. A correctly done underwater phase generally incorporates pushing off the wall, good streamlining and initiating transition at the appropriate time, but we only focused on the kinematics of the first component (by analysing the final push-off velocity, tuck index and foot plant index). Consequently, an integrative analysis combining the kinematic and kinetic characteristics of pushing off the wall associated with the turn-out strategy hydrodynamic variables should be incorporated in future research. Our age group swimmers were pooled for evaluation (in consideration of a reasonable sample size) with gender differences not being considered. We feel that swimmers should be analyzed by sex in future research, particularly if samples with swimmers after puberty are used. Last but not least, a control group should be added in future studies, allowing for the minimization of random effects on dependent variables over time and obtaining stronger experimental research designs.

## 5. Conclusions

The hydrodynamic characteristics (such as S, D and C_D_), as well as the pull-out strategy, were similar in the age group swimmers, irrespective of the backstroke-to-breaststroke turning technique used. Taken together with previous recommendations available in the literature, these findings highlight that optimizing propulsion during push-off, the glide depth, limb actions during transition (without decreasing velocity) and distance and time optimization could directly influence turn-out performance. We are confident that the current data is useful and will serve as a base for future studies centred on the relationship between biomechanical variables and how a change in hydrodynamic characteristics and pull-out strategy can provide a better understanding of the most efficient backstroke-to-breaststroke turns.

## Figures and Tables

**Table 1:** Kinematic variables selected to analyze the pull-out strategies of backstroke-to-breaststroke turning techniques.

Variables	Definition
Push-off velocity	Sacrum resultant velocity at the moment the feet left the wall.
Tuck index	Right hip distance from the wall at the beginning of push-off divided by the swimmer’s lower limb length.
Foot plant index	Foot plant depth on the wall at the beginning of push-off divided by the swimmer’s lower limb length.
First gliding distance	Sacrum distance from the moment the feet left the wall to the transition phase’s beginning.
First gliding time	Sacrum time from the beginning of the feet leaving the wall to the transition phase’s beginning.
First gliding depth	Sacrum average depth during the first gliding.
Transition distance	Sacrum distance from the initial hand separation or start of dolphin lower limb action until the upper limbs are extended at the body’s sides.
Transition time	Sacrum time from the initial hand separation or starting dolphin lower limb action until the upper limbs are extended at the body’s sides.
Transition gliding depth	Sacrum average depth during the transition phase.
Second gliding distance	Sacrum distance from the first frame of the upper limbs being extended at body’s sides to the instant the hands begin to move up from the body’s sides.
Second gliding time	Sacrum time from the first frame of the upper limbs being extended at the body’s sides to the instant the hands begin to move up from the body’s sides.
Second gliding depth	Sacrum average depth during the second gliding.
Breakout distance	Distance at which the head breaks the surface for the first time.
Breakout time	Time at which the head breaks the surface for the first time.
Average pull-out velocity	Average velocity from the moment the feet leave the wall to the head breaking the surface.
Time to 7.5 m	Time from the feet leaving the wall to the head reaching the 7.5 m mark.

**Table 2:** Mean ± SD of the hydrodynamic characteristics of the first and second glidings in the four studied backstroke-to-breaststroke turning techniques.

Variables	Turning Techniques					*p*	*ω* ^2^
	Open	Somersault	Bucket	Crossover	All		
V_1_^st^ (m·s^−1^)	1.31 ± 0.14	1.33 ± 0.13	1.32 ± 0.12	1.32 ± 0.15	1.32 ± 0.13	0.89	0.001
V_2_^nd^ (m·s^−1^)	1.31 ± 0.02	1.33 ± 0.02	1.32 ± 0.02	1.32 ± 0.02	1.32 ± 0.13	0.89	0.001
C_D1_^st^	−0.74 ± 0.16	−0.72 ± 0.27	−0.74 ± 0.30	−0.75 ± 0.14	−0.74 ± 0.22	0.90	0.001
C_D2_^nd^	−1.14 ± 0.44	−1.12 ± 0.34	−1.17 ± 0.47	−1.27 ± 0.51	−1.18 ± 0.45	0.17	0.01
D_1_^st^ (N)	−36.73 ± 9.99	−39.01 ± 13.48	−40.28 ± 9.75	−41.78 ± 7.10	−39.35 ± 0.67	0.08	0.02
D_2_^nd^ (N)	−61.65 ± 5.76	−64.13 ± 5.42	−62.25 ± 12.34	−69.73 ± 16.55	−64.44 ± 15.11	0.25	0.001

V_1_^st^: mean velocity of the first gliding curve; V_2_^nd^: mean velocity of the second gliding curve; C_D1_^st^: drag coefficient of the first gliding position; C_D2_^nd^: drag coefficient of the second gliding position; D_1_^st^: drag force of the first gliding position; D_2_^nd^: drag force of the second gliding position.

**Table 3:** Mean ± SD values, *p* values and effect sizes regarding the kinematics and pull-out variables of four turning techniques of the backstroke-to-breaststroke turns.

Variables	Turning Techniques					*p*	ω^2^
	Open	Somersault	Bucket	Crossover	All		
Push-off velocity (m·s^−1^)	2.03 ± 0.04 ^c^	2.02 ± 0.05 ^c^	2.01 ± 0.04 ^c^	2.17 ± 0.05 ^o,s,b^	2.06 ± 0.03	0.01	0.05
Tuck index	0.71 ± 0.14 ^s^	0.76 ± 0.10 ^o^	0.76 ± 0.09	0.72 ± 0.12	0.74 ± 0.12	0.09	0.00
Foot plant index	0.59 ± 0.02 ^c^	0.68 ± 0.03 ^b,c^	0.55 ± 0.03 ^s^	0.50 ± 0.02 ^o,s^	0.58 ± 0.19	0.01	0.11
First gliding distance (m)	2.41 ± 0.56	2.60 ± 0.64	2.48 ± 0.58	2.44 ± 0.63	2.47 ± 0.65	0.07	0.02
First gliding time (s)	1.21 ± 0.42	1.34 ± 0.49	1.32 ± 0.38	1.29 ± 0.3.78	1.28 ± 0.45	0.18	0.01
First gliding depth (m)	0.56 ± 0.13 ^s^	0.73 ± 0.13 ^o,b,c^	0.57 ± 0.13 ^s^	0.57 ± 0.13 ^s^	0.61 ± 0.15	0.01	0.25
Transition distance (s)	1.09 ± 0.20	1.08 ± 0.22	1.10 ± 0.14	1.09 ± 0.19	1.09 ± 0.02	0.75	0.00
Transition time (s)	0.99 ± 0.22	0.92 ± 0.19	0.97 ± 0.16	0.96 ± 0.18	0.96 ± 0.19	0.23	0.01
Transition gliding depth (m)	0.62 ± 0.14 ^s^	0.86 ± 0.17 ^o,b,c^	0.67 ± 017 ^s^	0.65 ± 0.16 ^s^	0.70 ± 0.20	0.01	0.29
Second gliding distance (m)	0.78 ± 0.27	0.82 ± 0.34	0.86 ± 0.27	0.85 ± 0.32	0.85 ± 0.28	0.44	0.00
Second gliding time (s)	0.78 ± 0.03	0.83 ± 0.05	0.86 ± 0.05	0.85 ± 0.05	0.83 ± 0.30	0.19	0.01
Second gliding depth (m)	0.62 ± 0.17 ^s^	0.76 ± 0.17 ^o,b,c^	0.62 ± 0.16 ^s^	0.62 ± 0.15 ^s^	0.65 ± 0.18	0.01	0.14
Breakout distance (m)	5.97 ± 0.87	6.13 ± 0.94	6.05 ± 0.80	6.02 ± 0.91	6.04 ± 0.94	0.85	0.00
Breakout time (s)	4.84 ± 0.94	5.01 ± 0.96	4.83 ± 0.84	4.78 ± 0.91	4.86 ± 0.98	0.42	0.00
Average pull-out velocity (m·s^−1^)	1.06 ± 0.13	1.08 ± 0.14	1.08 ± 0.14	1.07 ± 0.12	1.07 ± 0.13	0.74	0.00
Time to 7.5 m (m)	7.19 ± 0.89	7.09 ± 0.91	7.06 ± 0.72	7.12 ± 0.82	7.12 ± 0.90	0.75	0.00

^o^, ^s^, ^b^ and ^c^: different from the open, somersault, bucket and crossover turns (respectively).

## Data Availability

Data presented in this study are available on request from the corresponding author. The data are not publicly available due to ethical reasons.
